# Autophagy Impairment in *App* Knock-in Alzheimer’s Model Mice

**DOI:** 10.3389/fnagi.2022.878303

**Published:** 2022-05-19

**Authors:** Richeng Jiang, Makoto Shimozawa, Johanna Mayer, Simone Tambaro, Rakesh Kumar, Axel Abelein, Bengt Winblad, Nenad Bogdanovic, Per Nilsson

**Affiliations:** ^1^Division of Neurogeriatrics, Department of Neurobiology, Care Sciences and Society, Center for Alzheimer Research, Karolinska Institutet, Solna, Sweden; ^2^Department of Otolaryngology Head and Neck Surgery, The First Hospital of Jilin University, Changchun, China; ^3^Department of Biosciences and Nutrition, Karolinska Institutet, Huddinge, Sweden; ^4^Theme Inflammation and Aging, Karolinska University Hospital, Huddinge, Sweden; ^5^Division of Clinical Geriatrics, Department of Neurobiology, Care Sciences and Society, Center for Alzheimer Research, Karolinska Institutet, Huddinge, Sweden

**Keywords:** Alzheimer’s disease, *APP* knock-in mice, autophagy, amyloid beta, protein homeostasis, p62, LC3, electron microscopy

## Abstract

Alzheimer’s disease (AD) is characterized by impaired protein homeostasis leading to amyloid-β peptide (Aβ) amyloidosis. Amyloid precursor protein (APP) knock-in mice exhibit robust Aβ pathology, providing possibilities to determine its effect on protein homeostasis including autophagy. Here we compared human AD postmortem brain tissue with brains from two different types of *App* knock-in mice, *App*^*NL*–*F*^ and *App*^*NL*–*G*–*F*^ mice, exhibiting AD-like pathology. In AD postmortem brains, p62 levels are increased and p62-positive staining is detected in neurons, including potential axonal beadings, as well as in the vasculature and in corpora amylacea. Interestingly, p62 is also increased in the neurons in 12-month-old *App*^*NL*–*G*–*F*^ mice. In brain homogenates from 12-month-old *App*^*NL*–*G*–*F*^ mice, both p62 and light chain 3 (LC3)-II levels are increased as compared to wildtype (WT) mice, indicating inhibited autophagy. Double immunostaining for LC3 and Aβ revealed LC3-positive puncta in hippocampus of 24-month-old *App*^*NL*–*F*^ mice around the Aβ plaques which was subsequently identified by electron microscopy imaging as an accumulation of autophagic vacuoles in dystrophic neurites around the Aβ plaques. Taken together, autophagy is impaired in *App* knock-in mice upon increased Aβ pathology, indicating that *App* knock-in mouse models provide a platform for understanding the correlation between Aβ and autophagy.

## Introduction

Alzheimer’s disease (AD) is the major form of dementia, leading to cognitive impairment. Extracellular amyloid-β (Aβ) peptide depositions and intracellular tau aggregation are the two pathological hallmarks found in the AD brain parenchyma. Aβ may also accumulate in the vasculature and lead to cerebral amyloid angiopathy (Lendahl et al., 2019). In addition to Aβ and tau pathologies, autophagy is impaired in AD (Nixon et al., 2005). Macroautophagy (herein referred to as autophagy) is a major intracellular degradative pathway and plays a crucial role in the metabolism of Aβ (Yu et al., 2005; Nilsson et al., 2013; Nilsson and Saido, 2014). In AD brains, an accumulation of autophagosomes accompanied by increased levels of lysosomal proteases is observed (Boland et al., 2008). Furthermore, previous studies have shown an increase in autophagy-related genes in the early stage of AD, indicating an activation of autophagy (Lipinski, 2010; Lipinski et al., 2010), whereas it becomes impaired in the late stage of the disease (Nixon et al., 2005; Yu et al., 2005; Lee et al., 2011; Wolfe et al., 2013). A compromised autophagy in AD is further supported by downregulation of key autophagy proteins including FIP200, Rubicon, Atg5, and Atg16 (Heckmann et al., 2020).

Autophagy is initiated from the phagophore that is induced by cargo receptors like sequestosome 1/p62 and NBR1 (Johansen and Lamark, 2011). In the p62-dependent autophagy pathway, p62 binds to microtubule-associated protein 1 light chain 3 (LC3) and participates in the formation of autophagosomes which have double membrane structures. p62 delivers cargos to be degraded by autophagy and is degraded by the lysosome (Pankiv et al., 2007). During autophagosome formation, LC3-I is conjugated with phosphatidylethanolamine to form LC3-II (Hemelaar et al., 2003; Tanida et al., 2004). LC3-II is therefore a specific autophagosome associated marker (Kabeya et al., 2000) and both p62 and LC3-II are commonly used for measuring autophagic flux (Tanida et al., 2008).

*App* knock-in mice exhibit robust Aβ pathology whereas *App* expression is at physiological levels because gene expression of the mouse *App* gene is under the control of the endogenous mouse *App* promoter. In these mice, two clinical mutations found in familial AD (FAD) have been inserted to the mouse *App* gene, the Swedish (NL) and the Beyreuther/Iberian (F) mutations. This leads to an increased generation of Aβ42 in the *App*^*NL*–*F*^ line, inducing an onset of Aβ amyloidosis at nine months of age and cognitive impairment at 18 months of age (Saito et al., 2014). In *App*^*NL*–*G*–*F*^ mice, the Arctic mutation (G) was additionally introduced, which leads to a more aggressive Aβ pathology starting at two months of age and memory impairment at six months of age (Saito et al., 2014). This aggressive Aβ pathology of *App*^*NL*–*G*–*F*^ mice drives earlier and more severe neuroinflammation and synaptic alteration as compared to *App*^*NL*–*F*^ mice (Saito et al., 2014).

In the current study, we analyzed two key autophagy markers p62 and LC3 in the two *App* knock-in mice, and the data indicate that *App*^*NL*–*G*–*F*^ mice are, at least to some extent, similar to the late stage of AD in terms of autophagy alterations. Moreover, electron microscopy (EM) analysis shows a significant autophagic vacuole accumulation, specifically around the Aβ plaques in 24-month-old *App*^*NL*–*F*^ mice.

## Methods

### Human Brain Samples

Human brain slides were provided by the brain bank of Karolinska Institutet (approval nr 2013/1301-31/2) (Supplementary Table 1). p62 immunoblotting was performed in brain homogenates from the Netherlands Brain Bank under ethical permit Dnr EPN 2011/962-31/1 and 2018/1993-32.

### Animals

*App*^*NL*–*F*^ and *App*^*NL*–*G*–*F*^ knock-in mice have been described previously (Saito et al., 2014). *App*^*NL*–*F*^ mice contain the Swedish (KM670/671NL) and the Beyreuther/Iberian (I716F) mutations whereas *App*^*NL*–*G*–*F*^ mice have additionally the Arctic (E693G) mutation inserted into the mouse *App* gene (Saito et al., 2014). *App* knock-in mouse experiments were performed under ethical permit ID 407 approved by Linköping animal ethical board and 12570-2021 approved by Stockholm animal ethical board. Mice were kept on 12:12 light–dark cycle and with *ad libitum* access to food.

### Mouse Brain Dissection

The mice were anesthetized by isoflurane. Thereafter, the mice were perfused with phosphate buffered saline (PBS) through cardiac perfusion and the mouse brains were collected. One hemisphere of the brain tissue was fixed in 10% formalin solution (Merck Millipore, MA, United States, Cat. HT501128) for immunohistochemistry, and the other hemisphere was dissected into hippocampus and cortex for biochemical analysis.

### Primary Neuron Culture

Six-well plates were coated by poly-D-lysine (Sigma-Aldrich, MO, United States, cat. P6407) for 1 h at room temperature and washed three times with Milli-Q water. Mouse brains from E17 embryos from WT and *App*^*NL*–*G*–*F*^ female mice were separated in Hanks’ balanced salt solution (HBSS) (Thermo Fisher Scientific, MA, United States, Cat. 14175095) and cortex/hippocampus were dissected under the dissection microscope. Cortex/hippocampus were transferred to a falcon tube and HBSS was removed. Mixture of 97% Neurobasal Medium (Thermo Fisher Scientific, MA, United States, Cat. 21103049), 1% Glutamax (Thermo Fisher Scientific, MA, United States, Cat. 35050038), and 2% B-27 (Thermo Fisher Scientific, MA, United States, Cat. 17504044) were added and tissues were separated by pipetting 20–30 times. Cells were counted by using a hematocytometer after trypan blue staining and 4.5 × 10^5^ cells were seeded. After 18 DIV, cells were treated with 100 nM bafilomycin A1 (Sigma-Aldrich, MO, United States, Cat. B1793) for 6 h and then the cells were collected and lysed in radioimmunoprecipitation assay (RIPA) buffer (Thermo Fisher Scientific, MA, United States, Cat. 89901) containing protease and phosphatase inhibitors for 10 min. Cell lysate was sonicated for 1 min and centrifuged at 15,000 rpm at 4°C for 20 min. Supernatant was transferred to the new tubes for western blot analysis.

### Immunohistochemistry

For human brain samples, 3,3′-Diaminobenzidine (DAB) staining was performed with Dako EnVision Systems/HRP (Agilent Technologies, CA, United States, Cat. K401011-2). Slides were deparaffinized by xylene and ethanol and blocked by peroxidase (Dako kit) for 5 min and with 10% normal goat serum for 20 min at room temperature. The slides were incubated with anti-p62 antibody (Cell Signaling Technology, MA, United States, Cat. 5114) (1:500) in 3% normal goat serum overnight. After washing with 0.05% PBS-T buffer, slides were incubated with an anti-rabbit secondary antibody (Dako kit) for 30 min at room temperature, followed by incubating with chromogen solution, which was diluted in DAB substrate buffer (Dako kit), for 5 min at room temperature. Before mounting, slides were counterstained with Mayer’s Hematoxylin for 30 s and processed by dehydration. Images were acquired by the Nikon Eclipse E800 microscope with Nikon DS-Ri2 camera (acquisition parameters: 64 analog gain, 15-ms exposure time, and 4,908 × 3,264 pixel resolution) and were quantified by ImageJ software.

For mouse brain samples, paraffin-embedded brain tissues were sectioned into 4-μm thick sections. Antigen retrieval was performed, and after blocking, slides were incubated with anti-Aβ antibody (82E1) (Immuno-Biological Laboratories, Hokkaido, Japan, Cat.10323) (1:1,000), anti-LC3B (Novus Biologicals, CO, United States, Cat. NB100-2220) (1:2,000), and anti-p62 antibody (1:100). The following day, slides were incubated with secondary antibodies: alexa 546 goat anti-mouse (Invitrogen, MA, United States, Cat. A11030) (1:200), biotinylated goat anti-mouse IgG (Vector Laboratories, CA, United States, Cat. BA-9200) (1:200), and biotinylated goat anti-rabbit IgG (Vector Laboratories, CA, United States, Cat. BA-1000) (1:200). The biotinylated secondary antibodies were amplified with TSA Fluorescein System (PerkinElmer, MA, United States, Cat. NEL701001KT). Images were acquired by the Nikon Eclipse E800 microscope with Nikon DS-Qi2 camera (acquisition parameters: 64 analog gain, 500 ms or 1 s exposure time and 4,908 × 3,264 pixel resolution) and were quantified by ImageJ software.

### Western Blot

Fresh frozen mouse brain samples were homogenized in RIPA buffer supplemented with phosphatases inhibitors (Sigma-Aldrich, MO, United States, Cat. P0044) and proteases inhibitors (G-Biosciences, MO, United States, Cat. 786-433) or separated into cytosolic and membrane fractions. For cytosolic fraction, tissues were homogenized in 10 mM Tris (pH: 8.0) and 0.25 M sucrose and centrifuged at 4,000 rpm for 15 min at 4^°^C. Supernatant was centrifuged at 53,000 rpm for 1 h at 4^°^C. The supernatant was kept as a cytosolic fraction. Tissue homogenates and cell lysates were boiled at 95^°^C for 3 min except for analysis of Atg9A and Atg16L. A 20 μg portion of protein was loaded onto 4–20% SDS-PAGE for separation and transferred to polyvinylidene difluoride (PVDF) or nitrocellulose membranes. The membranes were blocked by 5% skim milk and were probed by primary antibodies, anti-p62 (1:500), anti-LC3 (Novus Biologicals, CO, United States, Cat. NB100-2331) (1:1,000), anti-p-Ulk1 S555 (Cell Signaling Technology, MA, United States, Cat. 5869) (1:500), anti-p-Ulk1 S757 (Cell Signaling Technology, MA, United States, Cat. 14202) (1:500), anti-Ulk1 (Cell Signaling Technology, MA, United States, Cat. 8054) (1:500), anti-Atg5 (Novus Biologicals, CO, United States, Cat. NB110-53818) (1:500), anti-Atg7 (Santa Cruz Biotechnology, CA, United States, Cat. sc-376212) (1:200), anti-Atg9A (Abcam, United Kingdom, Cat. ab108338) (1:500), anti-Atg16L (MBL Life Science, Tokyo, Japan Cat. PM040) (1:500), and anti-β-actin (Sigma-Aldrich, MO, United States, Cat. A2228) (1:10,000) overnight at 4^°^C. The next day, the PVDF or nitrocellulose membranes were incubated with fluorescently labeled secondary antibodies (LI-COR Biosciences, NE, United States), IRDye 800CW donkey anti-rabbit (Cat. 926-32213) (1:10,000), IRDye 680RD goat anti-rabbit (Cat. 926-68071) (1:10,000), IRDye 800CW donkey anti- mouse (Cat. 926-32212) (1:10,000), or IRDye 680RD goat anti-mouse (Cat. 926-68070) (1:10,000) for 1 h at room temperature. Images were acquired by a fluorescence imaging system (LI-COR Biosciences, NE, United States, Odyssey CLx) and were analyzed by Image Studio Lite (LI-COR Biosciences, NE, United States) software.

### Ribonucleic Acid Extraction, Complementary Deoxyribonucleic Acid Synthesis, and Real-Time Polymerase Chain Reaction

Fresh mouse brain tissue was kept in RNAprotect Tissue Reagent (Qiagen, Venlo, Netherlands, Cat. 76104) and Ribonucleic acid (RNA) was extracted according to the manufacturer’s instruction of RNeasy Lipid Tissue Mini Kit (Qiagen, Venlo, Netherlands, Cat. 74804). After measuring RNA concentration, 200 ng of RNA was used for complementary deoxyribonucleic acid (cDNA) synthesis according to manufacturer’s instructions of the High-Capacity cDNA Reverse Transcription Kit (Thermo Fisher Scientific, MA, United States, Cat. 4374966). The TaqMan Fast Advanced Master Mix (Thermo Fisher Scientific, MA, United States, Cat. 4444557) was used to perform the real-time polymerase chain reaction (RT-PCR) using TaqMan mouse gene expression assays (FAM) (Thermo Fisher Scientific, MA, United States, Cat. 4331182), Mm00448091_m1 for Sqstm1 (Gene aliases: p62). The gene expression level was normalized to TaqMan mouse gene expression assays (VIC) (Thermo Fisher Scientific, MA, United States, Cat. 4448489), Mm02619580_g1 for Atcb. Each sample was triplicated and run in the 7500 Fast Real-Time PCR System (Applied Biosystems, MA, United States).

### Transmission Electron Microscopy

Mice, 24-month-old, were anesthetized by isoflurane and perfused with 2.5% glutaraldehyde and 1% paraformaldehyde in 0.1 M phosphate buffer by cardiac perfusion. Thereafter, the mouse brains were collected and fixed in the same solution as used in perfusion process. Brain tissues were sectioned coronally into 1-mm thickness on the brain slicer matrix. The coronal brain slices were further dissected under the dissection microscope to separate the hippocampus. The hippocampal tissues were rinsed in 0.1 M phosphate buffer prior to the post fixation in 2% osmium tetroxide in 0.1 M phosphate buffer at 4^°^C for 2 h. Tissues were dehydrated stepwise in ethanol and acetone and eventually embedded in LX-112. Prior to the ultrathin sections, the tissue slices were mounted on the glass slides and stained with a toluidine blue solution to visualize the hippocampal area. Ultrathin sections were prepared using an EM UC7 (Leica, Wetzlar, Germany) and the grids contrasted with uranyl acetate and lead citrate. Aβ42 was expressed and purified from *Escherichia coli* (Abelein et al., 2020). Aβ42 fibril was prepared by incubating 3 μM Aβ42 monomer at 37^°^C for 24 h. After fibril formation, 10 μl of Aβ42 fibril was spotted on copper grid coated with formvar/carbon. Excess sample was wiped with Whatman filter paper and then grid was washed two times with 10 μl of Milli-Q water. It was then stained with 1% uranyl formate and then air dried. The sample was then imaged using TEM. Imaging was performed under the Hitachi HT7700 transmission electron microscope (Hitachi High-Technologies, Tokyo, Japan) operated at 80 kV equipped with a 2*k* × 2*k* Veleta CCD camera (Olympus Soft Imaging Solutions, Münster, Germany).

### Statistical Analysis

For the analysis of biochemical data, one-way ANOVA followed by Dunnett’s multiple comparisons test was performed for the three-group comparisons while two-tailed Student’s *t*-test was performed for the two-group comparisons in GraphPad Prism 8.

## Results

### Autophagy Alterations in Alzheimer’s Disease Postmortem Brain

**FIGURE 1:**
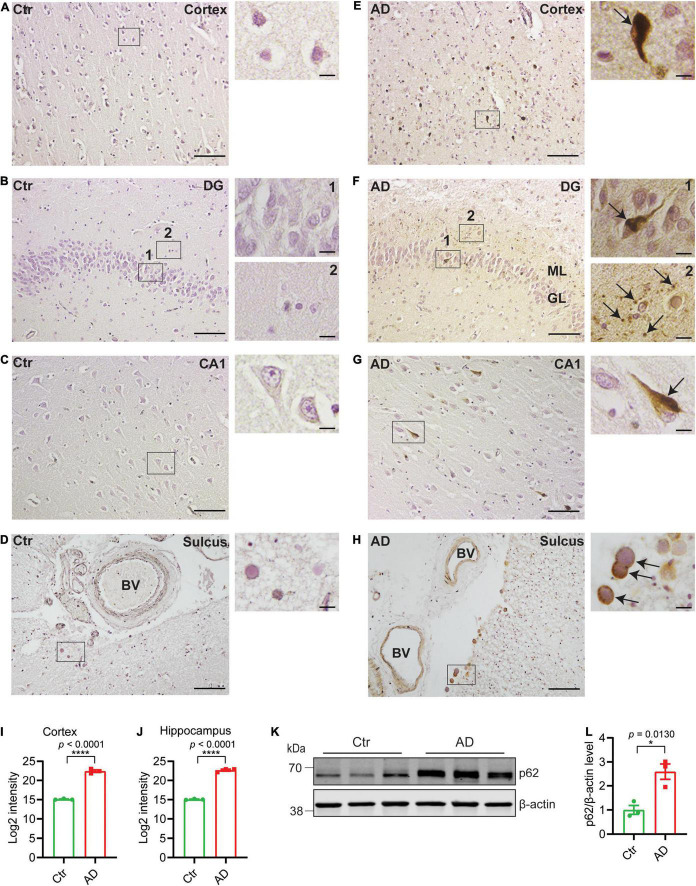
p62 accumulates in AD brains. Immunohistochemistry of p62 in entorhinal cortex, DG, and CA1 of hippocampus and sulcus as indicated from healthy control (Ctr) **(A–D)** and AD brains **(E–H)**. Scale bars represent 100 μm. Higher magnification images of depicted areas (black boxes) are shown to the right. Scale bars represent 10 μm. Arrows in **(E,F1,G)** indicate p62 accumulation in neurons of entorhinal cortex and hippocampus; **(F2)** indicates potential axonal beadings in ML of DG; **(H)** indicates corpora amylacea. **(H)** p62 is accumulated in tunica intima of the blood vessels in AD. **(I)** Semiquantitative density measurement was performed for quantification; 20× magnification images of six different and separate regions of cortex were chosen from each individual and the intensities were quantified and presented as log2-transformed data. **(J)** 20× magnification images of DG, CA1, and CA3 (two for each region) were chosen for hippocampus and the intensities were quantified and presented as log2-transformed data (*n* = 3 patients/group, ****p < 0.0001). **(K)** Western blot analysis of p62 in human postmortem prefrontal cortex from AD and healthy control subjects and **(L)** quantified by densitometry (*n* = 3 patients/group, *p < 0.05). Data were analyzed by Student’s *t*-test. Data are represented as mean ± SEM. DG, dentate gyrus; BV, blood vessel; ML, molecular layer; GL, granular layer.

To investigate autophagy alteration in AD postmortem brain, we stained for p62. p62 is normally degraded along with the cargo during functional autophagy and therefore p62 accumulation and aggregation indicate impaired autophagy. We immunostained brains from healthy controls (Figures 1A–D) and AD subjects (Figures 1E–H) and quantified the p62-positive signals. In AD brains, p62 accumulation occurs in pyramidal neurons as previously shown (Kuusisto et al., 2002) in both cortex (Figure 1E) and hippocampus including dentate gyrus (DG) (Figure 1F) and CA1 (Figure 1G) of AD brains (semi-quantified in Figures 1I,J), whereas no p62 accumulation was found in brains of non-demented individuals (Figures 1A–C). p62 accumulated inside the neurons (Figures 1E,G arrow in depicted area and Figure 1F arrow in depicted area 1) and was also identified as small round puncta (Figure 1F arrows in depicted area 2). The puncta may represent axonal beadings, which are a series of swellings along the axons located in the molecular layer of DG. Interestingly, tunica intima of the vessels and corpora amylacea also contains substantial amount of p62 whereas no staining was observed in healthy controls (Figures 1D,H). Western blot analysis of brain homogenates from AD subjects with Braak stages 5–6 revealed significantly increased p62 levels as compared to healthy subjects (Figures 1K,L). In summary, p62 accumulation was frequently observed in AD brains, which indicates a disturbed autophagy-lysosomal system potentially caused by an inhibition of autophagy in both neurons and cells in the tunica intima of vessels.

### Autophagy Alterations in *App* Knock-in Mice

**FIGURE 2:**
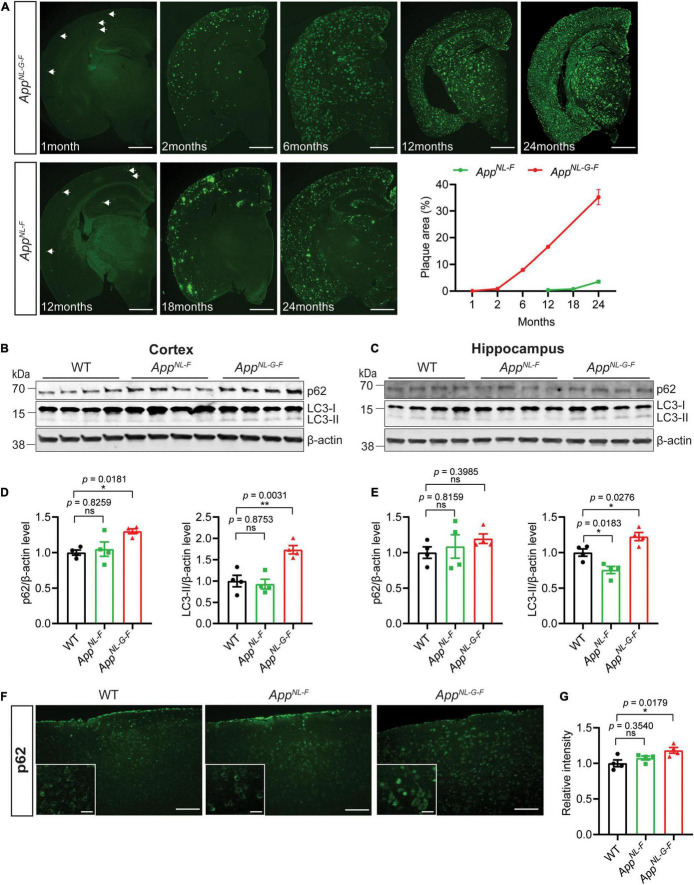
Autophagy alterations in *App* knock-in mice. **(A)** Immunostaining for Aβ plaque deposition in the brains of *App*^*NL*–*F*^ and *App*^*NL*–*G*–*F*^ mice of indicated ages. The percentage of Aβ plaque area in the whole brain was quantified. Scale bars represent 1,000 μm. **(B)** Cytosolic fraction of cortical and **(C)** hippocampal brain homogenates from 12-month-old WT, *App*^*NL*–*F*^, and *App*^*NL*–*G*–*F*^ mice were immunoblotted with autophagic markers p62 and LC3. **(D)** The levels of p62 and LC3-II in cortex and **(E)** hippocampus were quantified by densitometry (*n* = 4, *p < 0.05, **p < 0.01). **(F)** Immunostaining of p62 in 12-month-old WT, *App*^*NL*–*F*^, and *App*^*NL*–*G*–*F*^ mouse cortex. Scale bars represent 100 μm. The zoom in images of p62 positive neurons were inserted in white box. Scale bars represent 20 μm. **(G)** The relative intensities were quantified (*n* = 4, *p < 0.05). Data were analyzed by one-way ANOVA followed by Dunnett’s multiple comparisons test. Data are represented as mean ± SEM. ns, not significant.

To investigate the effect of the increasing Aβ amyloidosis in the *App* knock-in mice (Figure 2A) on autophagy, we analyzed markers of autophagic flux. The *App* knock-in mice exhibited alterations in autophagy as shown by western blot analysis for autophagy markers LC3 and p62 using 12-month-old WT, *App*^*NL*–*F*^, and *App*^*NL*–*G*–*F*^ mouse brains. *App*^*NL*–*F*^ mice started to accumulate Aβ plaques at 12 months of age whereas *App*^*NL*–*G*–*F*^ mice exhibited a very pronounced Aβ pathology at this age (Figure 2A). We found that p62, LC3-II, and LC3-II/LC3-I ratio were significantly increased in the cortex of *App*^*NL*–*G*–*F*^ mice (Figures 2B,D and full blot of p62 in Supplementary Figure 1A, higher exposure of LC3 blots in Supplementary Figure 1B, and LC3-II/LC3-I in Supplementary Figure 1C) whereas p62 mRNA level was unaltered (Supplementary Figure 1E), indicating that autophagy was inhibited in the cortex of *App*^*NL*–*G*–*F*^ mice. The LC3-II levels were also similarly increased in the hippocampus of *App*^*NL*–*G*–*F*^ mice, although the ratio of LC3-II/LC3-I did not change significantly (Figures 2C,E and higher exposure time of LC3 blots in Supplementary Figure 1B, and LC3-II/LC3-I in Supplementary Figure 1D). In agreement with the p62 western blot data, immunostaining showed a significant accumulation of p62 in the cortex of *App*^*NL*–*G*–*F*^ mice as compared to WT mice (Figures 2F,G) whereas both p62 and LC3-II levels are unaltered in mouse primary neurons from *App*^*NL*−*G*−*F*^ mice as compared to WT (Supplementary Figures 1F–J). In contrast to *App*^*NL*–*G*–*F*^ mice, the LC3-II levels were significantly decreased in the hippocampus of *App*^*NL*–*F*^ mice whereas no changes of p62 levels in cortex and hippocampus were detected (Figures 2B–G).

**FIGURE 3:**
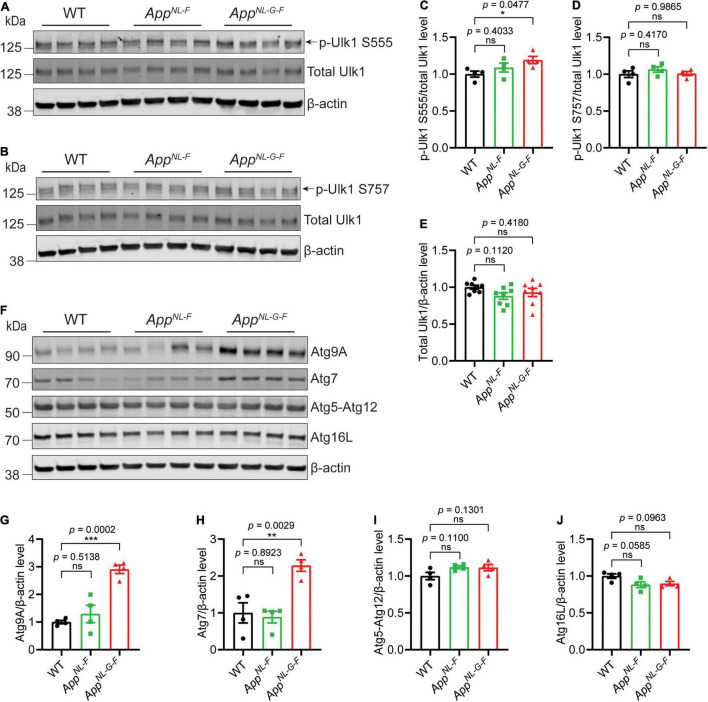
Increased levels of key autophagy proteins in *App*^*NL*–*G*–*F*^ mice. Western blot analysis of cytosolic fraction of cortical brain homogenates from 12-month-old *App*^*NL*–*F*^ and *App*^*NL*–*G*–*F*^ mice were analyzed for Ulk1, p-Ulk1 S555 and p-Ulk1 S757 **(A–E)**, Atg9A, Atg7, Atg5-Atg12, Atg16L **(F–J)**, and the levels were quantified by densitometry (*n* = 4, *p < 0.05, **p < 0.01, ***p < 0.001). Data are represented as mean ± SEM. Ns, not significant.

Previous studies have identified alterations in several key autophagy proteins in AD brains (Heckmann et al., 2020). We therefore next analyzed the brains of the *App* knock-in mice for these proteins. The data interestingly showed a significant increase of p-Ulk1 S555 specifically in *App*^*NL*–*G*–*F*^ mouse cortex, which is involved in activating initiation of autophagy; whereas no change of p-Ulk1 S757, which is involved in inhibition of autophagy initiation, was observed (Figures 3A–E). In addition, a significant increase of both Atg7 and Atg9A was detected in the brains of *App*^*NL*–*G*–*F*^ mice whereas Atg5–Atg12 and Atg16L were unaltered (Figures 3F–J).

**FIGURE 4:**
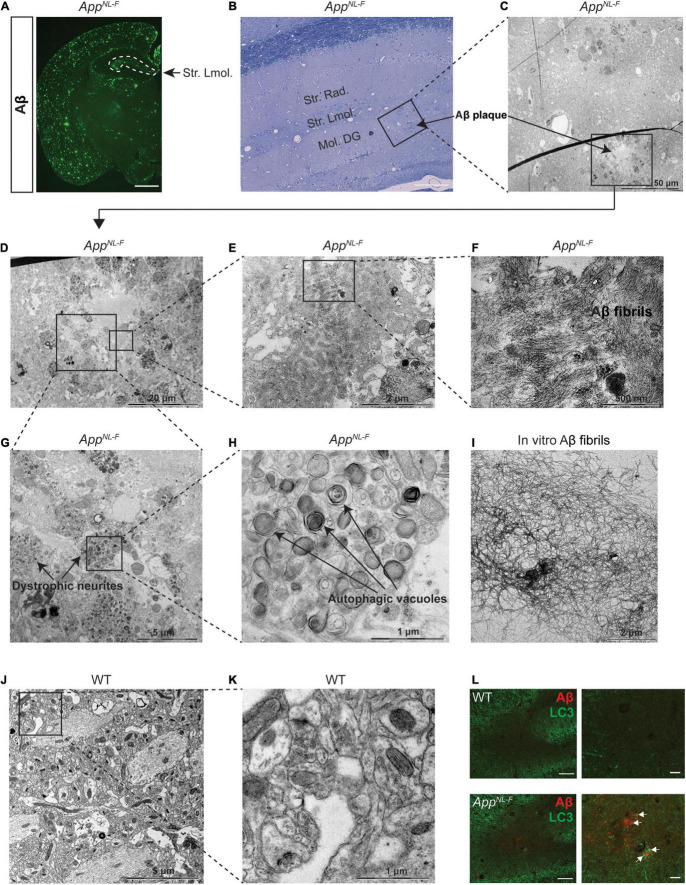
Autophagic vacuole accumulation in dystrophic neurites in aged *App*^*NL*–*F*^ mice. **(A)** Immunostaining of Aβ plaques in 24-month-old *App*^*NL*–*F*^ mouse brains. Scale bars represent 1,000 μm. **(B)** Toluidine blue staining of 24-month-old *App*^*NL*–*F*^ mouse hippocampus. Scale bars represent 100 μm. The area labeled in black box was imaged under electron microscopy (EM) and shown in **(C)**. The area in black box in **(C)** showing Aβ plaques was zoomed in **(D)**. The area in black boxes in **(D)** were further zoomed in and shown in **(E,G)**, showing Aβ plaques and dystrophic neurites, respectively. Aβ plaques and fibrils were shown in **(F)** and the autophagic vacuoles were shown by higher magnification in **(H)**. **(I)** Aβ fibrils formed *in vitro* and imaged by EM. **(J)** The EM image from 24-month-old WT mouse hippocampus and the area in the black box was zoomed in **(K)**. Scale bars for EM images were presented in the images. **(L)** Double immunostaining of Aβ and LC3 in the hippocampus in 24-month-old WT and *App*^*NL*–*F*^ mice. Scale bars represent 100 μm. Str. Lmol., stratum lacunosum-moleculare; Str.Rad., stratum radiatum; Mol. DG, moleculare dentate gyrus.

To further investigate the autophagy alterations upon aging of the *App*^*NL*–*F*^ mice, we next performed western blot analysis of LC3 and p62 using 18- and 24-month-old *App*^*NL*–*F*^ mice and no significant differences were observed, which was also confirmed with p62 immunostaining (Supplementary Figures 2A–J). However, EM analysis of 24-month-old *App*^*NL*–*F*^ mice exhibiting robust Aβ pathology (Figure 4A), including the region of striatum lacunosum-moleculare layer (Figures 4B,C), revealed a significant accumulation of autophagic vacuoles in the dystrophic neurites, around the Aβ plaques consisting of Aβ fibrils (Figures 4D–I) whereas no Aβ plaques or dystrophic neurites containing autophagic vacuoles were found in 24-month-old WT mouse hippocampus (Figures 4J,K). In agreement with the EM data, double immunostaining of Aβ and LC3 further confirmed the presence of LC3-positive puncta in association with the Aβ plaques in the 24-month-old *App*^*NL*–*F*^ mice (Figure 4L). Taken these data together indicate that the autophagic system of *App*^*NL*–*G*–*F*^ mice is characterized by an increase of both p62 and LC3-II, indicating that the autophagic system is inhibited, whereas the *App*^*NL*–*F*^ mice exhibited a region-specific alteration around the Aβ plaques. Hence, the degree of Aβ pathology correlates with the extent of autophagy alterations in the *App* knock-in mice.

## Discussion

In this study, we aimed at investigating autophagy status in *App* knock-in mice and compare the pathology to human AD postmortem samples. Autophagy is altered in most neurodegenerative diseases, which share the common feature of dysfunctional protein homeostasis leading to aberrant protein aggregation. Both genetic and biochemical evidence for a dysfunctional autophagy-lysosomal system in AD is compelling (Kabeya et al., 2000; Wong and Cuervo, 2010). An accumulation of autophagosomes paralleled with increased levels of lysosomal proteases in brain tissues indicates that the autophagic activity is upregulated early in the disease while it at later stages is impaired (Nixon et al., 2005; Yu et al., 2005; Boland et al., 2008; Lee et al., 2011; Wolfe et al., 2013). FAD-linked mutation in presenilin 1 also leads to impaired acidification due to reduced vATPase activity (Lee et al., 2010). Accumulation of autophagy adaptor protein p62 is an indicator of impaired autophagy which colocalizes with phosphorylated tau in AD postmortem brains (Kuusisto et al., 2002). Here we further substantiate these findings by showing that p62 also accumulates in tunica intima of vessels, indicating autophagy alteration in the vasculature in the AD brain. In addition, p62 accumulates in corpora amylacea. Though of largely unknown function, they could be related to debris clearance, for instance, from degenerating neurons. To further investigate the relationship between Aβ amyloidosis and autophagy, taking into account that autophagy plays a key role in Aβ metabolism (Yu et al., 2005; Nilsson et al., 2013), we analyzed the brains of *App* knock-in mice. These mouse models exhibit robust Aβ42 pathology, neuroinflammation, and synaptic alterations with physiological levels of amyloid precursor protein (APP) due to the knock-in strategy of FAD mutations. Thereby, potential artifacts caused by APP overexpression employed in APP transgenic mice can be circumvented. Interestingly, we observed, similar to AD brains, an inhibition of autophagy as indicated by an increase of p62 and LC3-II in the *App*^*NL*–*G*–*F*^ mice, especially in the cortex wherein the Aβ pathology starts at an earlier age as compared to hippocampus. Interestingly, we also found an increase in p-Ulk1 S555 and in Atg7 and Atg9A, indicating that an increase in autophagy initiation is also present in *App*^*NL*–*G*–*F*^ mouse brain potentially as a compensatory effect of the heavy Aβ burden and impaired end-stage clearance present in the 12-month-old *App*^*NL*–*G*–*F*^ mice. However, it remains to be established if this effect is present in the neurons or in other cell types of the brain such as astrocytes or microglia. In contrast, the brains of *App*^*NL*–*F*^ mice are characterized by a slight decrease in LC3-II levels. Since no activation of autophagy as measured by p-Ulk1/Ulk1 nor any increase in key autophagy proteins was observed, the decrease in LC3-II could be linked to increased lysosomal clearance which needs further analysis. Even though the two *App* knock-in models share the Swedish and the Beyreuther mutations leading to increase Aβ42 generation, the *App*^*NL*–*G*–*F*^ mice additionally exhibit the Arctic mutation that induces a fast oligomerization of the Aβ peptide and an early and robust Aβ plaque deposition. It is tempting to speculate that the autophagy inhibition observed in the 12-month-old *App*^*NL*–*G*–*F*^ mice is due to the much-pronounced Aβ pathology; however, it cannot be excluded that the difference in autophagy status is a direct effect of the Arctic mutation on the autophagy-lysosomal system. In support of an effect on autophagy by an increased Aβ burden is the very pronounced accumulation of autophagic vacuoles specifically located around the Aβ plaques in the 24-month-old *App*^*NL*–*F*^ mice. The impaired autophagy may in turn induce aggregation of intracellular Aβ and potentially cause a vicious cycle and further contribute to the increased Aβ pathology.

## Data Availability Statement

The original contributions presented in the study are included in the article/Supplementary Material, further inquiries can be directed to the corresponding author/s.

## Ethics Statement

The studies involving human participants were reviewed and approved by approval nr 2013/1301-31/2 and EPN 2011/962-31/1 and 2018/1993-32. The patients/participants provided their written informed consent to participate in this study. The animal study was reviewed and approved by ethical permit ID 407 approved by Linköping animal ethical board and 12570-2021 approved by Stockholm animal ethical board.

## Author Contributions

RJ and PN initiated the project and designed the study. RJ, MS, JM, and ST conducted the biochemical experiments related to autophagy. RK and AA performed EM analysis of Aβ fibrils formed *in vitro.* RJ, NB, and BW analyzed human postmortem immunohistology. RJ, PN, and BW wrote the first draft of manuscript. NB advised on the study and commented on the manuscript. All authors reviewed and revised the manuscript.

## Conflict of Interest

The authors declare that the research was conducted in the absence of any commercial or financial relationships that could be construed as a potential conflict of interest.

## Publisher’s Note

All claims expressed in this article are solely those of the authors and do not necessarily represent those of their affiliated organizations, or those of the publisher, the editors and the reviewers. Any product that may be evaluated in this article, or claim that may be made by its manufacturer, is not guaranteed or endorsed by the publisher.

## References

[B1] Abelein A. Chen G. Kitoka K. Aleksis R. Oleskovs F. Sarr M. (2020). High-yield production of Amyloid-β peptide enabled by a customized spider silk domain. *Sci. Rep.* 10:235. 10.1038/s41598-019-57143-x 31937841 PMC6959368

[B2] Boland B. Kumar A. Lee S. Platt F. M. Wegiel J. Yu W. H. (2008). Autophagy induction and autophagosome clearance in neurons: relationship to autophagic pathology in Alzheimer’s disease. *J. Neurosci.* 28 6926–6937. 10.1523/JNEUROSCI.0800-08.2008 18596167 PMC2676733

[B3] Heckmann B. L. Teubner B. J. W. Boada-Romero E. Tummers B. Guy C. Fitzgerald P. (2020). Noncanonical function of an autophagy protein prevents spontaneous Alzheimer’s disease. *Sci. Adv.* 6:eabb9036. 10.1126/sciadv.abb9036 32851186 PMC7428329

[B4] Hemelaar J. Lelyveld V. S. Kessler B. M. Ploegh H. L. (2003). A single protease, Apg4B, is specific for the autophagy-related ubiquitin-like proteins GATE-16, MAP1-LC3, GABARAP, and Apg8L. *J. Biol. Chem.* 278 51841–51850. 10.1074/jbc.M308762200 14530254

[B5] Johansen T. Lamark T. (2011). Selective autophagy mediated by autophagic adapter proteins. *Autophagy* 7 279–296. 10.4161/auto.7.3.14487 21189453 PMC3060413

[B6] Kabeya Y. Mizushima N. Ueno T. Yamamoto A. Kirisako T. Noda T. (2000). LC3, a mammalian homologue of yeast Apg8p, is localized in autophagosome membranes after processing. *EMBO J.* 19 5720–5728. 10.1093/emboj/19.21.5720 11060023 PMC305793

[B7] Kuusisto E. Salminen A. Alafuzoff I. (2002). Early accumulation of p62 in neurofibrillary tangles in Alzheimer’s disease: possible role in tangle formation. *Neuropathol. Appl. Neurobiol.* 28 228–237. 10.1046/j.1365-2990.2002.00394.x 12060347

[B8] Lee J.-H. Yu W. H. Kumar A. Lee S. Mohan P. S. Peterhoff C. M. (2010). Lysosomal proteolysis and autophagy require presenilin 1 and are disrupted by Alzheimer-Related PS1 mutations. *Cell* 141 1146–1158. 10.1016/j.cell.2010.05.008 20541250 PMC3647462

[B9] Lee S. Sato Y. Nixon R. A. (2011). Lysosomal proteolysis inhibition selectively disrupts axonal transport of degradative organelles and causes an alzheimer’s-like axonal dystrophy. *J. Neurosci.* 31 7817–7830. 10.1523/JNEUROSCI.6412-10.2011 21613495 PMC3351137

[B10] Lendahl U. Nilsson P. Betsholtz C. (2019). Emerging links between cerebrovascular and neurodegenerative diseases-a special role for pericytes. *EMBO Rep.* 20:e48070. 10.15252/embr.201948070 31617312 PMC6831996

[B11] Lipinski M. M. (2010). Towards the global understanding of the autophagy regulatory network. *Autophagy* 6 1218–1220. 10.4161/auto.6.8.13772 20953147

[B12] Lipinski M. M. Zheng B. Lu T. Yan Z. Py B. F. Ng A. (2010). Genome-wide analysis reveals mechanisms modulating autophagy in normal brain aging and in Alzheimer’s disease. *Proc. Natl. Acad. Sci. U S A.* 107 14164–14169. 10.1073/pnas.1009485107 20660724 PMC2922576

[B13] Nilsson P. Saido T. C. (2014). Dual roles for autophagy: degradation and secretion of Alzheimer’s disease Abeta peptide. *Bioessays* 36 570–578. 10.1002/bies.201400002 24711225 PMC4316186

[B14] Nilsson P. Loganathan K. Sekiguchi M. Matsuba Y. Hui K. Tsubuki S. (2013). Aβ secretion and plaque formation depend on autophagy. *Cell Rep.* 5 61–69. 10.1016/j.celrep.2013.08.042 24095740

[B15] Nixon R. A. Wegiel J. Kumar A. Yu W. H. Peterhoff C. Cataldo A. (2005). Extensive involvement of autophagy in Alzheimer disease: an immuno-electron microscopy study. *J. Neuropathol. Exp. Neurol.* 64 113–122. 10.1093/jnen/64.2.113 15751225

[B16] Pankiv S. Clausen T. H. Lamark T. Brech A. Bruun J. A. Outzen H. (2007). p62/SQSTM1 binds directly to Atg8/LC3 to facilitate degradation of ubiquitinated protein aggregates by autophagy. *J. Biol. Chem.* 282 24131–24145. 10.1074/jbc.M702824200 17580304

[B17] Saito T. Matsuba Y. Mihira N. Takano J. Nilsson P. Itohara S. (2014). Single App knock-in mouse models of Alzheimer’s disease. *Nat. Neurosci.* 17 661–663. 10.1038/nn.3697 24728269

[B18] Tanida I. Ueno T. Kominami E. (2004). LC3 conjugation system in mammalian autophagy. *Int. J. Biochem. Cell Biol.* 36 2503–2518. 10.1016/j.biocel.2004.05.009 15325588 PMC7129593

[B19] Tanida I. Ueno T. Kominami E. (2008). LC3 and Autophagy. *Methods Mol. Biol.* 445 77–88. 18425443 10.1007/978-1-59745-157-4_4

[B20] Wolfe D. M. Lee J. H. Kumar A. Lee S. Orenstein S. J. Nixon R. A. (2013). Autophagy failure in Alzheimer’s disease and the role of defective lysosomal acidification. *Eur. J. Neurosci.* 37 1949–1961. 10.1111/ejn.12169 23773064 PMC3694736

[B21] Wong E. Cuervo A. M. (2010). Autophagy gone awry in neurodegenerative diseases. *Nat. Neurosci.* 13 805–811. 10.1038/nn.2575 20581817 PMC4038747

[B22] Yu W. H. Cuervo A. M. Kumar A. Peterhoff C. M. Schmidt S. D. Lee J. H. (2005). Macroautophagy - a novel beta-amyloid peptide-generating pathway activated in Alzheimer’s disease. *J. Cell Biol.* 171 87–98. 10.1083/jcb.200505082 16203860 PMC2171227

