# Supplementary material for: Autophagy Impairment in App Knock-in Alzheimer’s Model Mice
Source: Front Aging Neurosci. 2022 May 19;14:878303. doi: 10.3389/fnagi.2022.878303 (PMC9160569; doi:10.3389/fnagi.2022.878303)
Supplement: Supplementary file 1 [file Table_1.pdf]

Supplementary Material

Supplementary Table 1. List of postmortem brain samples used in immunohistochemistry.

Subjects	Gender	Age	Braak stage	CAA[1]	APOE[2]
Control 1	M	78	0	-	E3/E2
Control 2	M	78	0-I	-	Unknown
Control 3	M	51	0	-	Unknown
AD 1	F	78	VI	+	E3/E4
AD 2	F	78	VI	+	E3/E4
AD 3	M	52	VI	+	E3/E4

[1]CAA: cerebral amyloid angiopathy; [2]APOE: apolipoprotein E.
