# Supplementary material for: Autophagy Impairment in App Knock-in Alzheimer’s Model Mice
Source: Front Aging Neurosci. 2022 May 19;14:878303. doi: 10.3389/fnagi.2022.878303 (PMC9160569; doi:10.3389/fnagi.2022.878303)
Supplement: Supplementary file 2 [file Image_1.pdf]

## Supplementary Figure 1

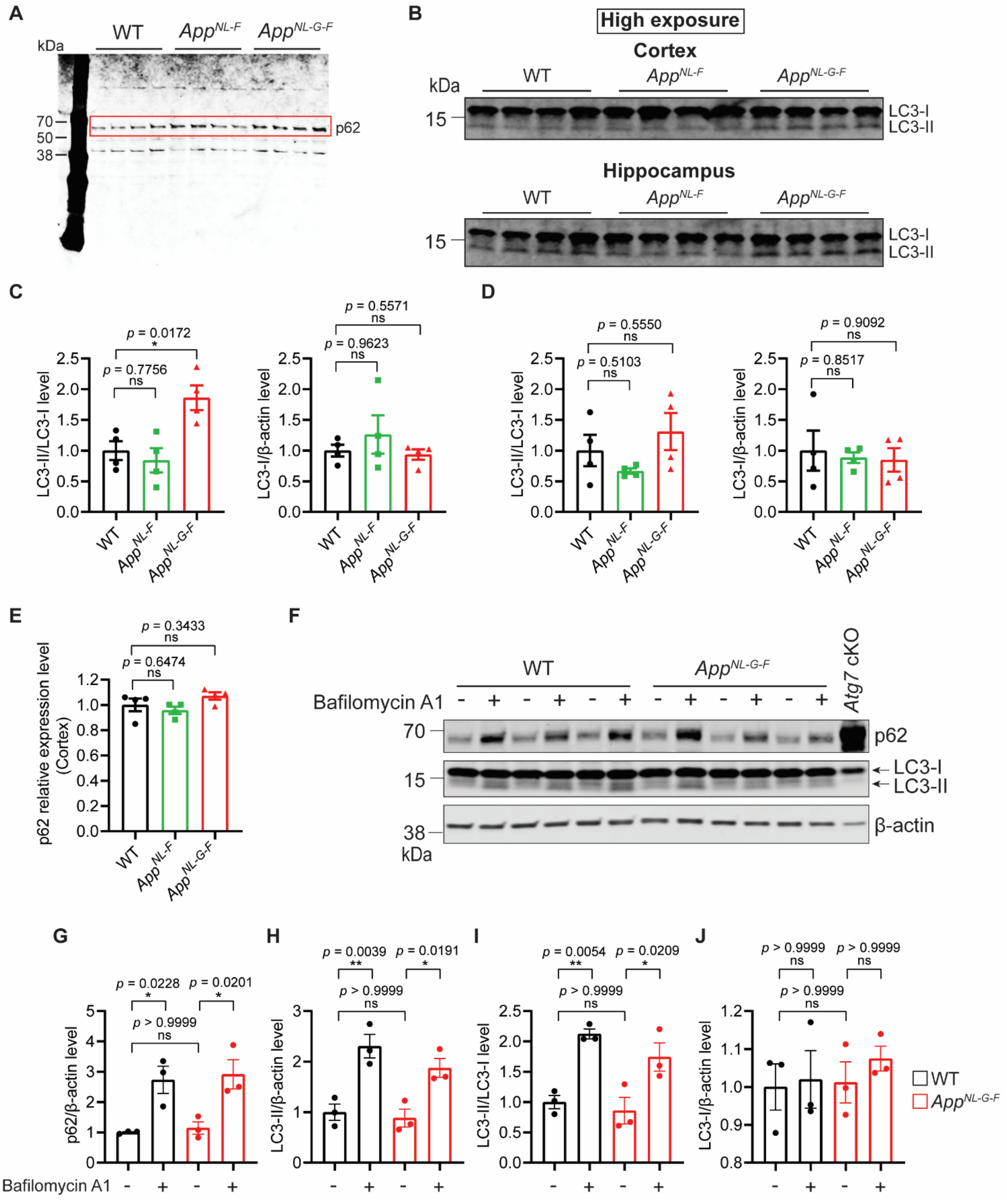

**Supplementary Figure 1.** ***No autophagy alteration in primary neurons from $App^{NL\text{-}G\text{-}F}$ mice*****.** **(A)** Full blot of p62 for Figure 2B. **(B)** Higher exposure of LC3 western blot for Figure 2B and 2D. The levels of LC3-II/LC3-I and LC3-I in cortex **(C)** and hippocampus **(D)** for Figure 2B and 2C were quantified by densitometry. (n = 4, *$p < 0.05$). **(E)** Relative mRNA expression level of p62 in cortex analyzed by qPCR. (n = 4). **(F)** Western blot of p62 and LC3 levels in WT and $App^{NL\text{-}G\text{-}F}$ mouse primary neurons treated with bafilomycin A1. *Atg7* conditional knock-out mouse brain homogenate was loaded in the right most lane as a positive control for both p62 and LC3-II. **(G-J)** The levels of p62, LC3-II, LC3-II/LC3-I and LC3-I were quantified by densitometry. (n = 3 embryos/genotype). The experiment was repeated once. Data are represented as mean ± SEM. ns: not significant.
