# Supplementary material for: Autophagy Impairment in App Knock-in Alzheimer’s Model Mice
Source: Front Aging Neurosci. 2022 May 19;14:878303. doi: 10.3389/fnagi.2022.878303 (PMC9160569; doi:10.3389/fnagi.2022.878303)
Supplement: Supplementary file 3 [file Image_2.pdf]

Supplementary Figure 2

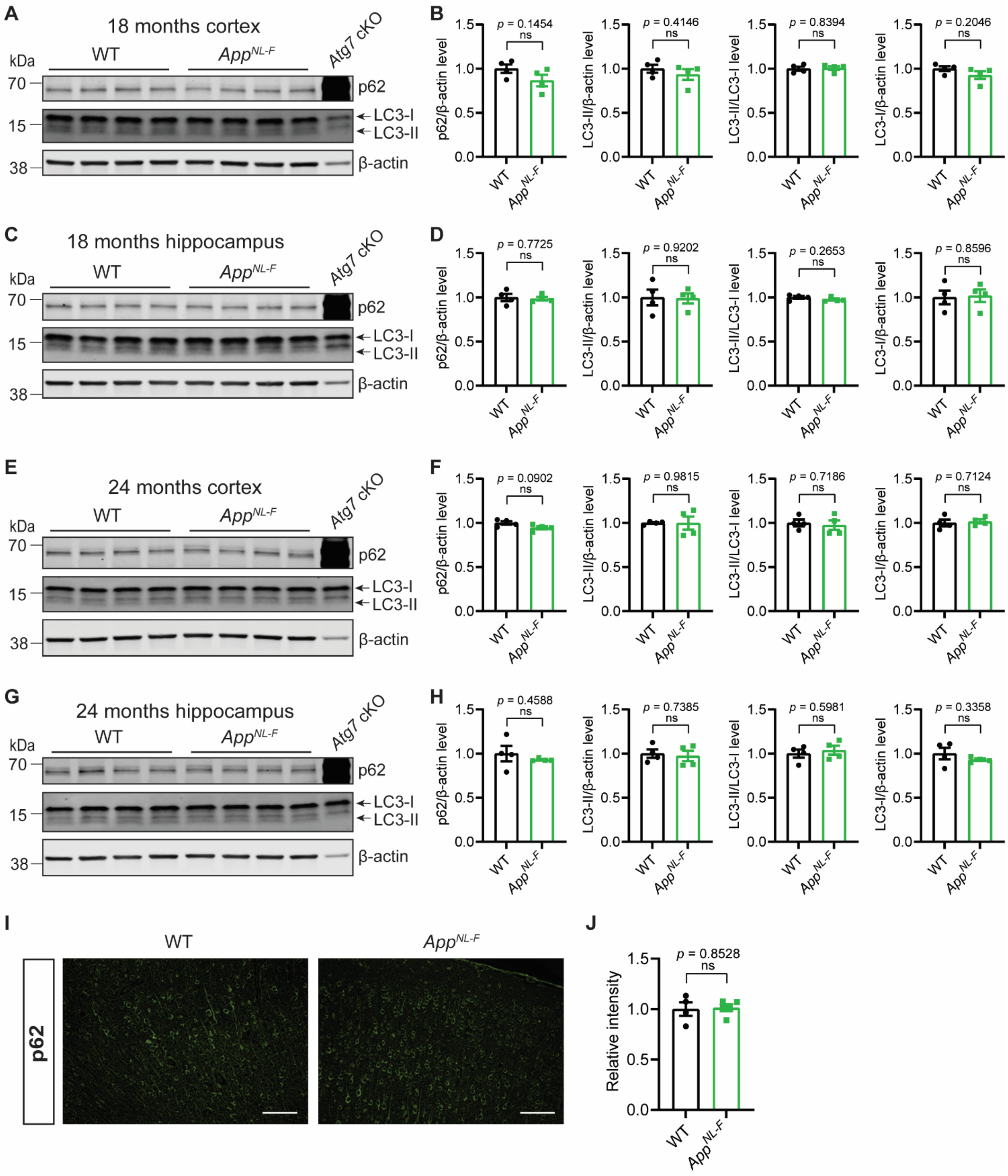

Supplementary Figure 2. ***No alterations of p62 and LC3 in aged App^{NL-F} mouse brains.*** **(A-H)** Quantitative western blot analysis for p62 and LC3 in cortical and hippocampal brain homogenates

from 18-months-old and 24-months-old WT and *App*$^{NL-F}$ mice. *Atg7* conditional knock-out mouse brain homogenate was loaded in the right most lane as a positive control for both p62 and LC3-II. (n = 4). **(I)** Immunostaining of p62 in 18 to 24-months-old WT and *App*$^{NL-F}$ mouse cortex. Scale bars represent 100 µm. **(J)** The relative intensities were quantified. (n = 4-5). Data are represented as mean ± SEM. ns: not significant.
